# Contrasting catastrophic eruptions predicted by different intrusion and collapse scenarios

**DOI:** 10.1038/s41598-018-24623-5

**Published:** 2018-04-18

**Authors:** M. Rincón, A. Márquez, R. Herrera, A. Alonso-Torres, J. L. Granja-Bruña, B. van Wyk de Vries

**Affiliations:** 1 Universidad Rey Juan Carlos, Área de Geología, Móstoles, Madrid, Spain; 2 Hospital Rey Juan Carlos, Radiodiagnóstico, Móstoles, Madrid, Spain; 3 Universidad Complutense, Dpto. Geodinámica, Estratigrafía y Paleontología, Madrid, Spain; 4 University of Clermont-Ferrand, Lab. Magmas et Volcans, Clermont-Ferrand, France

## Abstract

Catastrophic volcanic eruptions triggered by landslide collapses can jet upwards or blast sideways. Magma intrusion is related to both landslide-triggered eruptive scenarios (lateral or vertical), but it is not clear how such different responses are produced, nor if any precursor can be used for forecasting them. We approach this problem with physical analogue modelling enhanced with X-ray Multiple Detector Computed Tomography scanning, used to track evolution of internal intrusion, and its related faulting and surface deformation. We find that intrusions produce three different volcano deformation patterns, one of them involving asymmetric intrusion and deformation, with the early development of a listric slump fault producing pronounced slippage of one sector. This previously undescribed early deep potential slip surface provides a unified explanation for the two different eruptive scenarios (lateral vs. vertical). Lateral blast only occurs in flank collapse when the intrusion has risen into the sliding block. Otherwise, vertical rather than lateral expansion of magma is promoted by summit dilatation and flank buttressing. The distinctive surface deformation evolution detected opens the possibility to forecast the possible eruptive scenarios: laterally directed blast should only be expected when surface deformation begins to develop oblique to the first major fault.

## Introduction

Large stratovolcanoes are unstable structures liable to massive, catastrophic flank failures, with more than 20 historical well-documented cases since 1500 AD^1,2^ and about 200 in the last 10,000 years^3^. Historical volcanic landslides such as Bezymianny, Mount St Helens, Soufriere Hills and Tutupaca all generated large eruptions^2,4^, showing that when magma is involved, hazards multiply. Importantly, intruded magma creates strong deformation prior to landsliding^5,6^ and this can potentially be used to predict the type of eruption that follows^7^. The relationship between lateral collapse and magmatic eruption is known in deposits from a close association of debris avalanche deposits and pyroclastic products^8^. This shows that some intrusion-related landslides have triggered magmatic directed lateral blasts: the eruptive scenario known as a “Bezymianny-type” collapse^3^ (lateral collapse - lateral blast – vertical plinian eruption). However, in other cases collapse was followed only by a vertical plinian eruption^8^ (i.e. no lateral blast between the collapse and the plinian vertical jet).

The possibility of a landslide-related lateral blast has strong implications for hazards. Lateral blast absence has been attributed to failure occurring before magma intruded into the upper part of the edifice^8^. However, it is not clear what controls why in some cases, a volcanic edifice can collapse when the magma body is still located at the volcano base, nor is the relationship of magma-induced deformation to the collapse structure (e.g., the location and geometry of the slip surface) clear. Therefore, the motivation here is to understand the mechanisms that can explain why the two different eruptive scenarios (i.e., the existence/absence of a landslide-triggered lateral blast) can occur when a volcano collapses laterally during an intrusive episode.

In order to achieve that objective we model the structural evolution of a stratovolcano at the first stages of viscous magma intrusion in the edifice to find out what controls landsliding. We then use this information to find the conditions that determine the likelihood of blast or only vertical eruption on collapse.

We used physical analogue models as they can simulate the temporal evolution of discontinuous processes (i.e., faulting). We designed an experimental setup, based on previous work^9^, for scaled experiments of the intrusion of a viscous magma analogue (Golden syrup) at the base of an analogue stratovolcano made of a granular elasto-plastic material (sand-plaster mixture: see Methods). This kind of granular material deforms with both tension and shear fractures/faults and therefore is the most suitable for reproducing the mechanical behaviour of natural rocks^10^. Our experimental setup consists of a box where an 11 cm-high cone is made by pouring the sand-plaster mixture on the box floor above a sand-plaster layer 1 cm-thick. A tube with a tap inserted vertically at the box base allows introducing the magma analogue (Golden Syrup), which flows by gravity due to the height difference between the box bottom and a syrup reservoir attached to the tube. These experiments are designed to model the effect of viscous magma intrusion into a stratovolcano (see Methods), so they do not reproduce nor can be used to analyse other processes of deformation and volcano instability such as those related to the intrusion of fluid basaltic dykes in ocean island volcanoes^11^.

**Figure 1:**
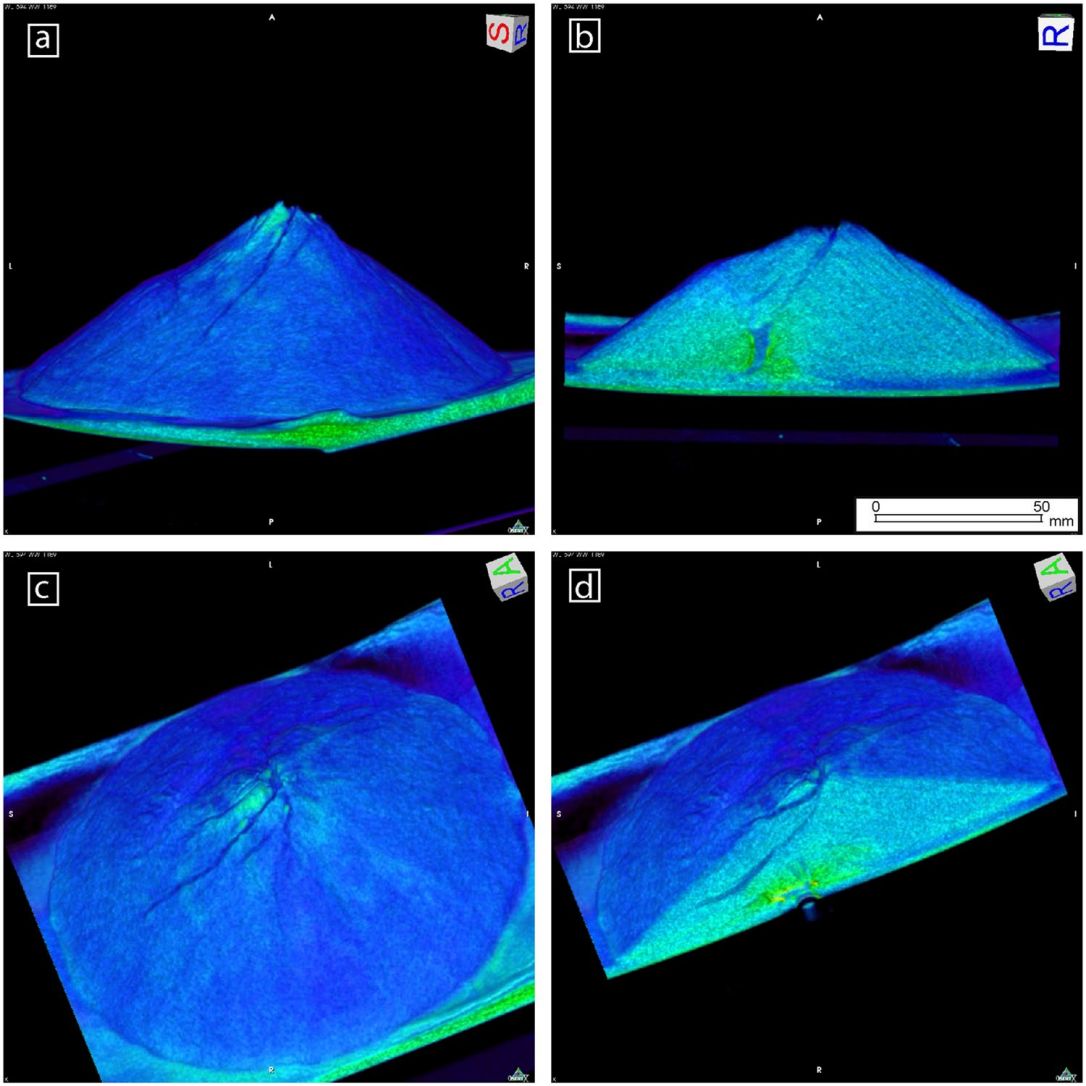
Examples of images of our experiments obtained by volumetric reconstruction from the MDCT data. (**a**) Lateral view of the cone surface showing the deformed topography of the left flank and the surface fracturing. (**b**) Cross section view showing the cone internal structure with the irregular syrup intrusion and faulting. Faults appear as darker bands as they are zones where density decreases by dilation. The Golden syrup intrusion is imaged as a homogeneous blue area. (**c**) 3D oblique view of the volcano surface showing fracturing. (**d**) Integrated surface and internal view (cross section) of the previous image 1c showing how the internal fracturing and surface volcano deformation can be correlated.

We made 44 similar experiments varying only the syrup flow rate and monitored 10 of them (Table 1) at Hospital Rey Juan Carlos using a Multiple Detector Computed Tomography (MDCT) scanner, an X-ray based technology (see Methods), to image the temporal evolution of intrusion-induced deformation, both at the surface and inside the volcano (Fig. 1). MDCT has been successfully used to monitor tectonic structures in analogue models^12^ but has not been used before to image such dynamic volcano intrusion experiments. We put our experimental setup on the mobile platform of the scanner, and before beginning the experiment we scanned our volcano in order to compare later images with the initial state (t0). During the experiment we scan the box again each 2 or 5 minutes, with the syrup flowing during the scanning procedure. We finished each experiment when syrup erupted at the volcano surface, obtaining experiment times between 12 and 50 minutes, although we focused in the results from the first 10–15 minutes, corresponding to the initial tens of days of the intrusion (see Methods). Developing our experiments over the scanner mobile platform, we have been able to obtain 3D data of the simulated volcano (Fig. 1), at several experiment temporal stages during intrusion. The data show a much more fine-scale detail of internal structures (Fig. 1) that obtained in previous experiments studied by means of sliced cross-sections^9^. By comparing results from different times, we can make 4D reconstructions of volcano internal faulting and intrusion together with surface deformation (Fig. 1). Additionally, volcano surface deformation has been quantified by extracting Digital Elevation Models of the cones from the MDCT data (see Methods) allowing us to detected and quantify temporal changes at volcano surface morphology (precision = 1 mm) which can be compared with volcano deformation data in natural cases.

**Table 1:** Experimental parameters and deformation patterns of the 10 experiments monitored by X-ray MDCT.

Experiment Number	Flow rate (cm^3^/min)	Distance tube center (cm)	Deformation pattern
9	2.0	1.8	1
13	3.1	0.8	1
14	1.3	1.0	1
5	1.3	3.5	2
6	1.5	2.6	2
7	1.0	2.5	2
10	1.1	2.7	2
16	3.1	3.6	2
4	2.5	2.7	3
15	1.6	2.3	3

## Results

### Deformation patterns

**Figure 2:**
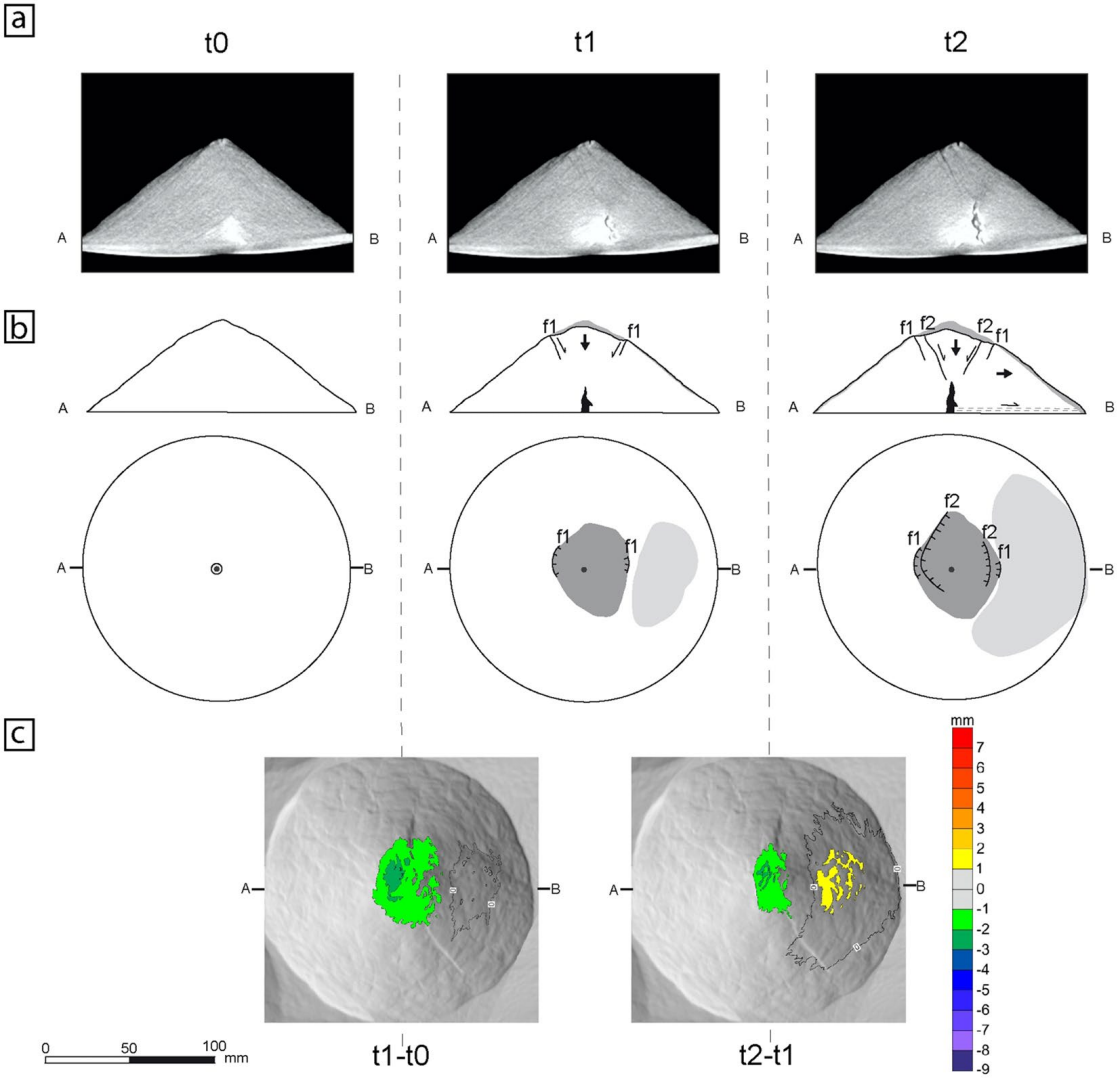
Structural evolution of deformation pattern 1. t0: stage before beginning the experiment; t1 and t2: stages at minutes 5 and 10 respectively. (**a**) X-ray cross-sections of the volcano (see b for sections location) of a representative experiment (Experiment 14: see Table 1). Faults are imaged as darker linear trends and syrup intrusion as a homogeneous dark grey area. Note the near-symmetric system of inward-dipping conjugate normal faults developed in the summit zone (fault dipping towards B being the most developed). (**b**) Sketched structural interpretation in cross-sections and map-view of the main deformation features common at experiments showing this deformation pattern (see text for an explanation). f1, f2: faults, in chronological order. Dashed lines: diffuse shear zones. Grey areas: changes at volcano topography (dark grey: subsidence; light grey: bulging). Black zones: magma bodies. Large arrows: movement of the volcano blocks (i.e., subsidence, lateral displacement or tilting). Thin arrows: relative movement along fault planes and basal shear zones. Circle: tube location. Dots: volcano centre. (**c**) Map-views showing the variation of the cone topography detected by MDCT between times t1 and t0 (t1-t0), and between t2 and t1 (t2-t1), over a shaded relief image of the cone. Contour interval: 1 mm. Colour scale at right. Note the subsidence of the summit area and the slight outward displacement of the flank opposite to the main summit fault.

**Figure 3:**
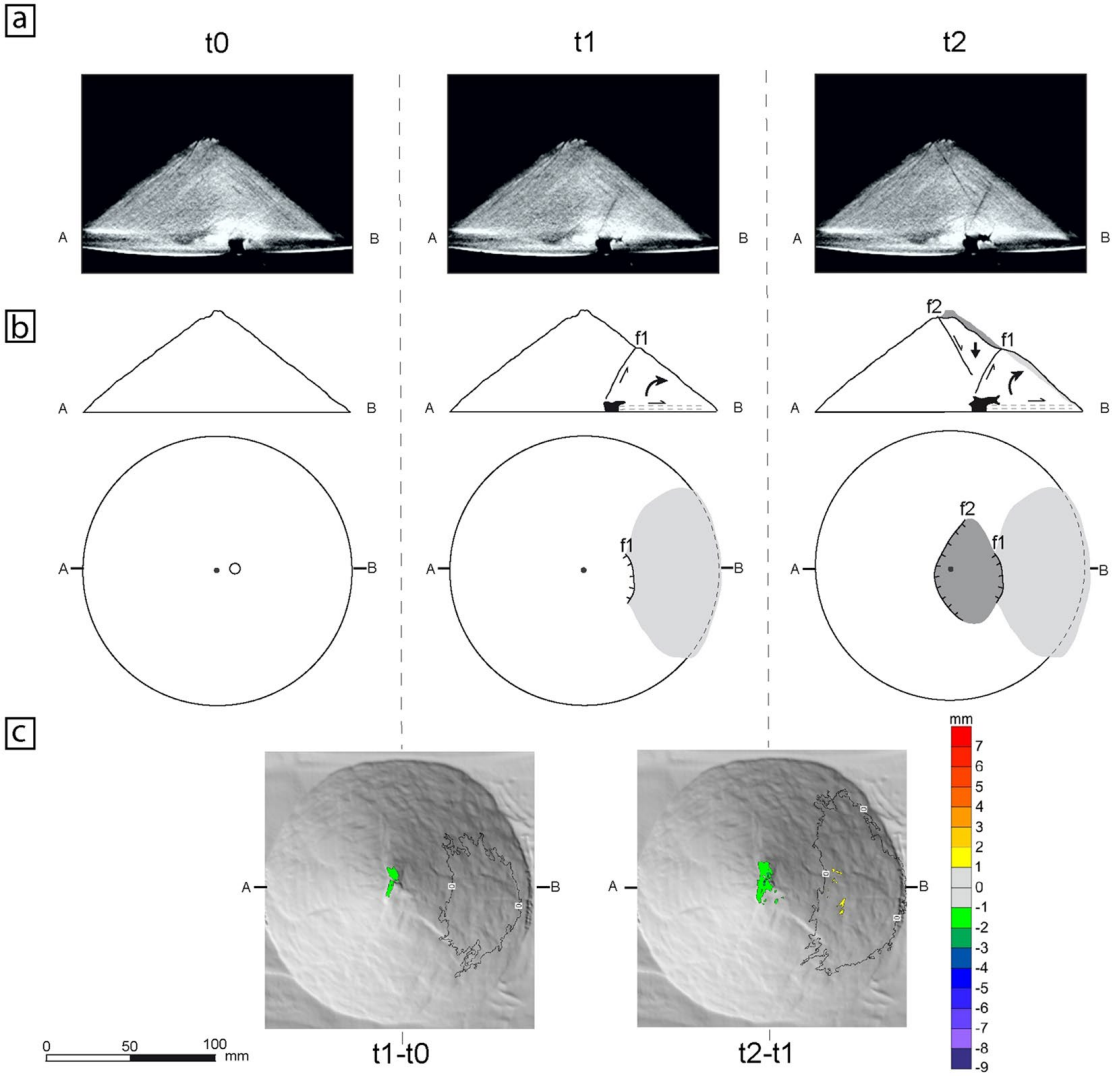
Structural evolution of deformation pattern 2. t0: stage before beginning the experiment; t1 and t2: stages at minutes 5 and 10 respectively. (**a**) X-ray cross-sections of the volcano (see b for sections location) of a representative experiment (Experiment 16: see Table 1). Faults are imaged as darker linear trends and syrup intrusion as a homogeneous dark grey area. Note at t1 image the development of a curved (i.e. listric) convex upward fault from the intrusive body towards the cone middle flank surface. At t2 image that fault has reached the cone surface and a new inward-dipping planar fault is developed from near the summit zone. (**b**) Sketched structural interpretation in cross-sections and map-view of the main deformation features common at experiments showing this deformation pattern (see text for an explanation). f1, f2: faults, in chronological order. Dashed lines: diffuse shear zones. Grey areas: changes at volcano topography (dark grey: subsidence; light grey: bulging). Black zones: magma bodies. Large arrows: movement of the volcano blocks (i.e., subsidence, lateral displacement or tilting). Thin arrows: relative movement along fault planes and basal shear zones. Circle: tube location. Dots: volcano centre. (**c**) Map-views showing the variation of the cone topography detected by MDCT between times t1 and t0 (t1-t0), and between t2 and t1 (t2-t1), over a shaded relief image of the cone. Contour interval: 1 mm. Colour scale at right. Note the slight subsidence of the summit zone together with the outward displacement of the lower flank downwards the first fault.

**Figure 4:**
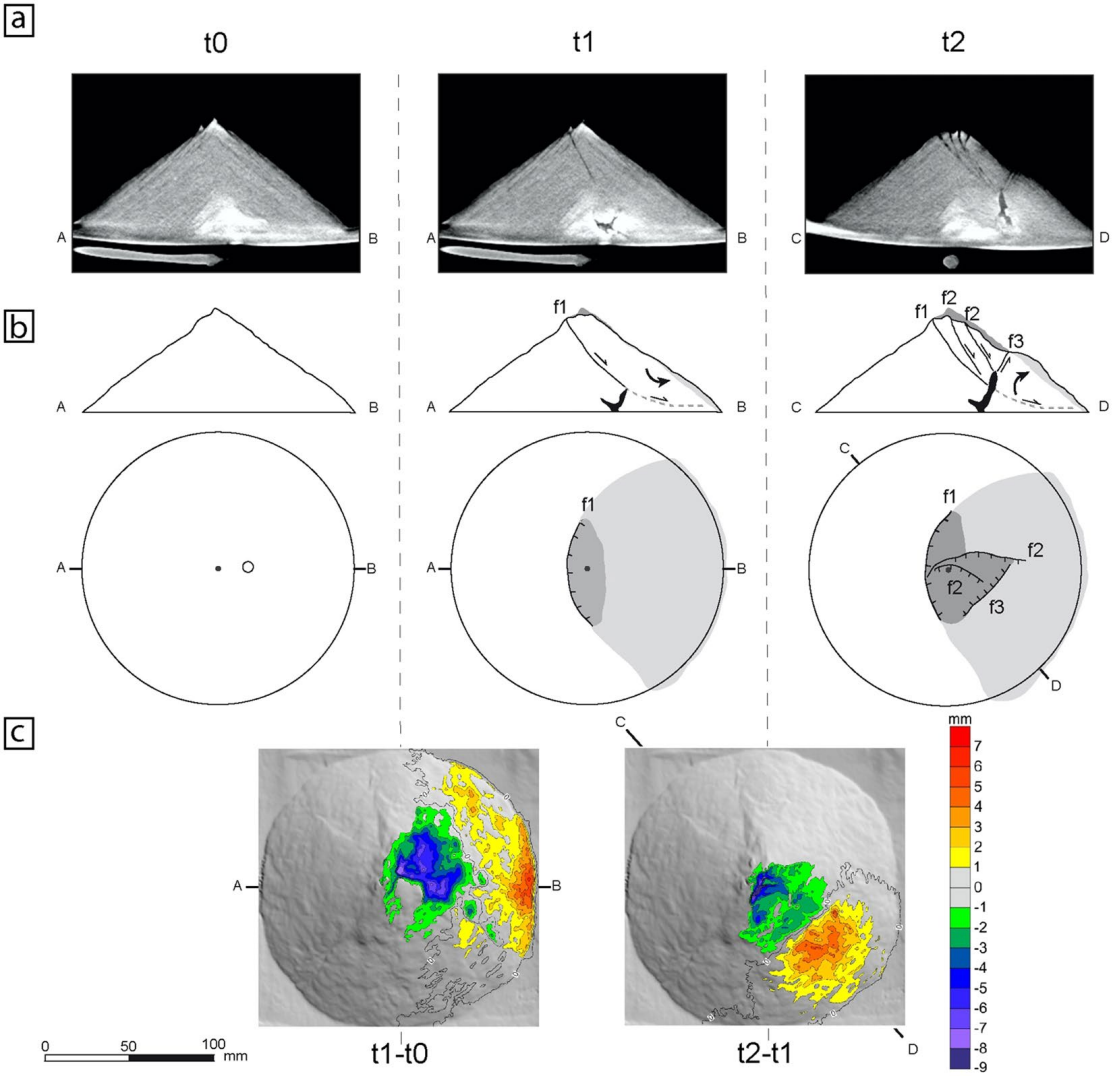
Structural evolution of deformation pattern 3. t0: stage before beginning the experiment; t1 and t2: stages at minutes 5 and 10 respectively. (**a**) X-ray cross-sections of the volcano (see b for sections location) of a representative experiment (Experiment 4: see Table 1). Faults are imaged as darker linear trends and syrup intrusion as a homogeneous dark grey area. Note at t1 image the development of an inward-dipping fault from near the cone summit. At t2 image a conjugate system of faults has developed near summit zone inside the displaced block. (**b**) Sketched structural interpretation in cross-sections and map-view of the main deformation features common at experiments showing this deformation pattern (see text for an explanation). f1, f2, f3: faults, in chronological order. Dashed lines: diffuse shear zones. Grey areas: changes at volcano topography (dark grey: subsidence; light grey: bulging). Black zones: magma bodies. Large arrows: movement of the volcano blocks (i.e., subsidence, lateral displacement or tilting). Thin arrows: relative movement along fault planes and basal shear zones. Circle: tube location. Dots: volcano centre. (**c**) Map-views showing the variation of the cone topography detected by MDCT between times t1 and t0 (t1-t0), and between t2 and t1 (t2-t1), over a shaded relief image of the cone. Contour interval: 1 mm. Colour scale at right. Note how at t1 the entire flank delimited by the first fault has been displaced, subsiding at the summit area and bulging at the lower flank. At t2 two complementary deformation zones have developed inside the displaced block, and oblique to the first trend: a summit subsidence zone and a lower bulge delimited by the inward-dipping fault visible at t2.

Deformation produced in all of our experiments can be classified into three different patterns based on the similarity of intrusion shapes and fault development (Figs 2, 3 and 4; Table 1). The first pattern (Fig. 2) is marked by a near-symmetric system of inward-dipping planar conjugate normal faults developed in the volcano summit zone. The summit sector, delimited by a set of faults (f1 and f2; Fig. 2b), subsides, together with a slight outward lateral displacement of one flank (Fig. 2b and c). In the transition from t1 to t2, the conjugate set fault f1 does not show any evidence of movement, but a new conjugate set fault f2 appears in the internal region of the summit sector. The main characteristic of the final deformation stage is the subsided summit sector (i.e. forming a graben like-structure; Fig. 2b). The intrusion has vertical near-cylindrical geometry and faulting begins when it reaches around one third of the volcano height (t1 at Fig. 2a and b). Three of the ten of our monitored experiments at MDCT x-ray scanner developed this deformation pattern (Table 1).

The second pattern is strongly different in both intrusion shape and faulting pattern (Fig. 3). Faulting initially develops an asymmetric nature: its main feature is the early listric (convex upwards) fault (f1 at Fig. 3b) which nucleates at the first stage close to the intrusive body and developed from bottom-to-top reaching the volcano surface at the middle-cone flank (Fig. 3b). A bulge is formed at the lower volcano flank, limited upwards by this f1 fault, because of clockwise tilting and outward lateral displacement of the lower flank (Fig. 3b). At a second stage, the fault f1 does not move and a new inward-dipping planar fault (f2) develops from near the summit on the opposite flank to the bulge (t2 at Fig. 3). The summit sector subsides as flank bulging progresses, whereas the flank sector down from the second fault (opposite to the bulge) remains undeformed (Fig. 3). The intrusion shape is different to cylindrical bodies of pattern 1: intrusion initially develops an irregular cup-shaped geometry^13^, with a later preferential growth into the bulging flank (Fig. 3a and b). This deformation pattern has been found in five of our x-ray scanned experiments (Table 1).

The third pattern (Fig. 4) is also asymmetrical, but it shows the early development from top-to-bottom of a large listric (concave upwards) fault (f1 at Fig. 4a and b) from near the summit to the base, and involving a substantial edifice volume. This fault shows a well-defined curved trend in the summit sector of the edifice and progressively fades towards deeper zones where connects with a diffuse basal shear zone (Fig. 4b). In some examples, this entire sector over the fault (i.e. the hanging-wall block) forms a rotational landslide, while the rest of edifice remains stable (Fig. 4c). The intrusion also shows an initial cup-shaped geometry and is located within the listric fault foot-wall block. At a second stage (t2 at Fig. 4), deformation changes and a new asymmetric conjugate fault system develops inside the unstable sector and oblique to the first fault (f1). Two listric (concave upwards) normal inward-dipping faults (f2) form from near the summit together with an opposite-dipping normal fault (f3) at the middle-cone flank (Fig. 4b, t2). The upper part of the intrusion is now located inside the sliding block delimited by f1 (i.e. in the hanging-block) and reaches the base of the conjugate fault system (f2 and f3). The surface sector affected by these new faults shows two different deformation zones delimited by the convex-upward fault f3: a subsided summit sector (between by faults f2 and f3) and a bulged lower zone (Fig. 4b, t2). In this deformation pattern, the volume of the edifice deformed and the magnitude of the deformation are significantly larger than in the 1^st^ and 2^nd^ cases (cf. Figs 2c, 3c and 4c). Two of our x-ray experiments have developed this deformation pattern (Table 1).

### Factors influencing deformation patterns

**Figure 5:**
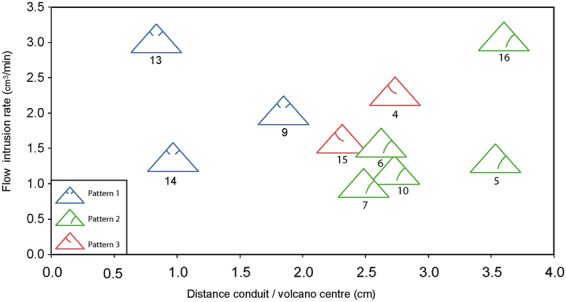
Influence of experimental parameters (Distance conduit/volcano centre vs. Flow intrusion rate) on the occurrence of the different deformation patterns. Experiments showing symmetrical deformation pattern 1 occur only when the volcano centre is aligned with the experimental conduit, whereas when the volcano construction is asymmetrical regarding the location of the syrup conduit, then deformation patterns at the volcanoes are also of asymmetrical nature (patterns 2 and 3). Intrusion flow rate does exert any influence on the deformation style.

The three different deformation patterns detected in our results are not related to the parameter that we varied systematically in our experimental set: the magma (syrup) flow rate. Experiments showing deformation patterns 1, 2 and 3 appear both at low flow rates (1–1.5 cm^3^/min) and at high flow rates (2.5–3 cm^3^/min; Table 1). However, after the analysis of the MDCT images of our experiments, we observed that during the construction processes of the cone by pouring the sand-plaster mixture, sometimes there was a small lateral offset between the centre of the cone and the tube (see for example Fig. 3a). This asymmetry (produced accidentally during the experiment construction) seems to be a key parameter controlling the occurrence of the different deformation patterns detected (Fig. 5): when the cone and the tube are vertically aligned (i.e., the distances between their centres are less than 2 cm) the cones deformation follows the symmetric pattern 1; whereas, when the cone and tubes centres are displaced (between 2 and 3.5 cm: Fig. 5), then the cones deformation follows the asymmetric patterns 2 or 3. Therefore, the initial asymmetry in the experimental setup induced the asymmetric deformation of the cone in response to the stresses induced by the fluid pressure. This role of asymmetry in influencing the deformation patterns at modelled volcanoes agrees with results from previous experiments of both intrusion and volcano spreading^14–16^.

With our experimental set we have not found any parameter (or combination of parameters; see Fig. 5) which apparently could be controlling the occurrence of deformation pattern 2 vs. 3 in an asymmetric experiment; i.e., why in some cases faulting developed first bottom-to-top from the intrusion (pattern 2) and in other cases the first fault developed top-to-bottom from the volcano near-summit surface (pattern 3). Other studies have shown that the construction of individual cones created very small heterogeneities and that intrusion experiments always have a wide range of outcomes^13,17^. For example, the role of bedding planes or layers with different strength for the development of large-scale sliding planes in the volcano has been noted in previous experiments^14^ and proposed for natural volcanoes where there is great heterogeneity^18^. The capability of X-ray MDCT scanning technique for a successful 4-D monitoring of volcano intrusion experiments shown in this work, clearly opens a very promising approach to decipher the early deformation stages, testing systematically the possible role of those asymmetries, and therefore may possibly finds the mechanism controlling the development of different asymmetrical deformation patterns.

## Discussion

**Figure 6:**
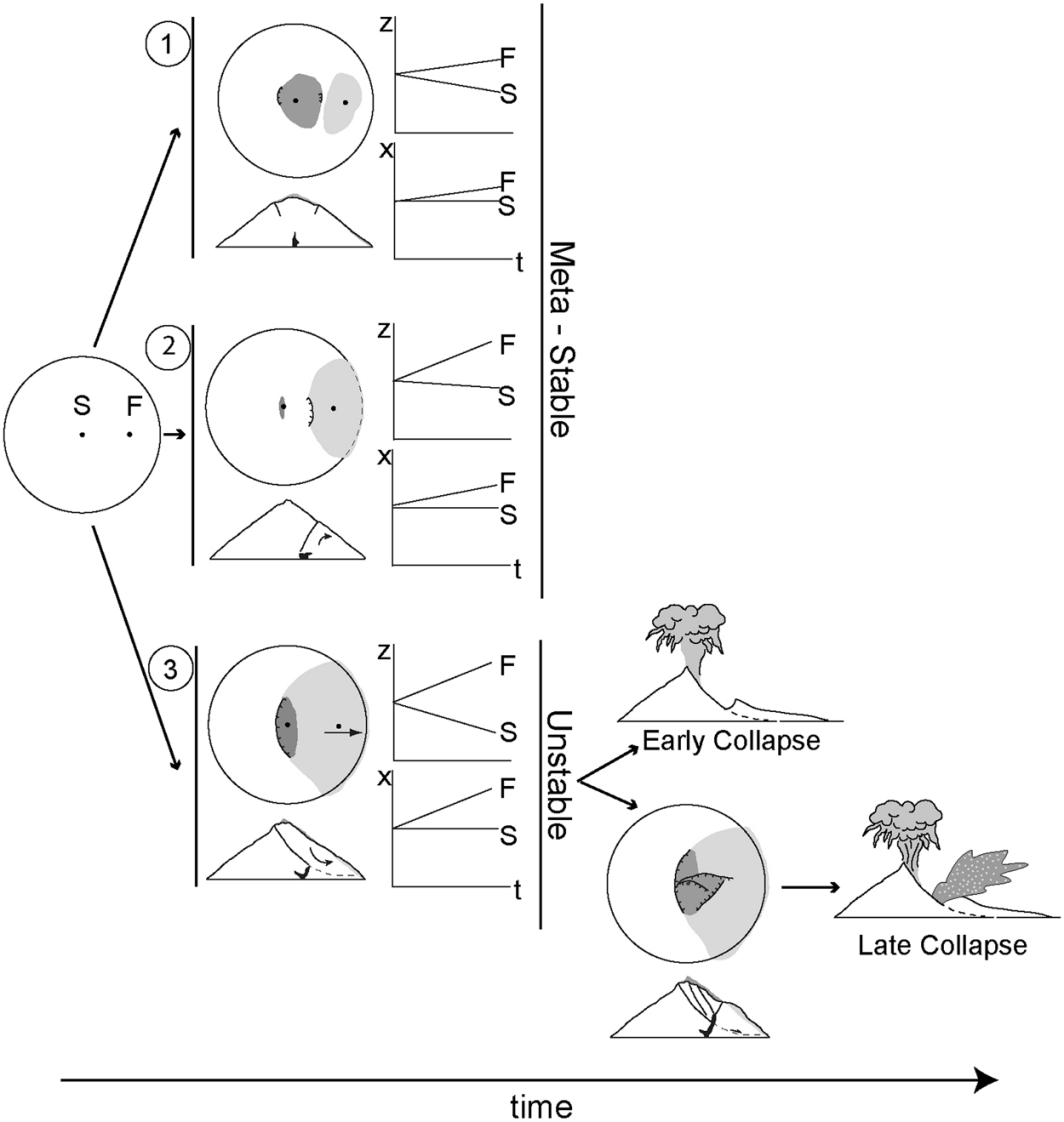
Event tree of the different volcano deformation patterns produce by the intrusion of a viscous magma body and the possible catastrophic eruptive scenarios in case of edifice lateral collapse. Map views show the deformation features (lines: faults; dark grey areas: subsidence; light grey areas: uplift) produced with each deformation pattern, together with the qualitative temporal behaviour of two hypothetical GPS stations located at the summit (S) and the flank (F) of the volcano continuously recording vertical (z) and horizontal (x) displacements. Sketched cross-sections show the development of deformation patterns 1 and 2 that do not produce deformation structures which induce the volcano instability, whereas deformation 3 produces a clear unstable situation. If deformation pattern 3 is detected, then two different eruptive scenarios can evolve in relation to the time of the volcano collapse. The “lateral collapse – plinian eruption” scenario is produced when the intrusive body is still located below the slip surface and therefore is not involved in the collapse (Early Collapse). “Bezymianny-type” (lateral collapse - lateral blast - plinian eruption) scenario is produced when part of the magmatic body has intruded inside the sliding block (Late Collapse). In late collapse there is dilation, then exposure of the magma body, producing a magmatic lateral directed blast.

The structural setting detected in pattern 3 experiments (Fig. 4) can explain the two different eruptive scenarios related to lateral volcano collapse (Fig. 6). The early development of the large listric fault (Fig. 4: t1) provides the main instability factor: a deep potential slip surface, which explains the deep-seated nature of volcano lateral collapses^19^. This large fault, forming a potential slip surface crossing the volcano, has not been previously noted, and explains why in some cases the volcanic edifice can collapse when the magma body is still located at the volcano base. In some experiments we observed how the entire flank slides on the fault as a large rotational landslide (Fig. 4c: surface deformation produced at t1-t0).

When a type-3 deformation pattern is developed, the temporal relationship between the collapse time and magma body position in relation to the fault explains the eruptive response: a blast only occurs if the magma has intruded into the sliding block. If any trigger induces the unstable sector failure at an earlier first stage, then the magmatic body would still be located below the collapse surface (Fig. 4: t1), and no juvenile material could be involved in the collapse. The unloading produced by such a large removal of volcano mass induces rapid shallow magma decompression and triggers its explosive eruption, producing a vertical plinian eruptive column (Fig. 6: Early Collapse). In the models, the area above the intrusion is more dilated, so would be more easily pierced by ascending, expanding magma.

If the catastrophic lateral collapse of the volcano flank occurs later, when magma has intruded into the sliding block (Fig. 4: t2), then as the landslide began magma would be rapidly decompressed. The magma would then be exposed in the first moments of sliding. Both decompression and exposure produce the lateral blast involving juvenile magmatic material (Fig. 6: Late Collapse).

The deformation observed at Mount St Helens at 1980 before its collapse^5^ strongly resembles to that observed in the time 2 of pattern 3 (Fig. 4: t2), with an elongated subsidence area at summit zone and a bulge at middle flank with similar shapes and extents. There was around 50 m of subsidence and bulging at St Helens^5^ and 5–6 mm at our model 4 (Fig. 4c: t2-t1) which scaled up is also about 50 m. This similar deformation at the stage 2 of our models fits with a scenario where the magma body has entered the sliding block. This concurs with the protracted strong surface deformation monitored (more than 45 days^5^) at Mount St Helens and the precursory vertical eruptions.

The detected deformation pattern thus provides a unified framework for the two different eruptive scenarios (lateral vs. vertical) with flank collapse. Early collapses when the magma body is still located at the base the volcano likely produce only a vertical eruption, whereas late collapses (i.e., when the magma body has penetrate in the sliding block) leads to lateral blast (Fig. 6).

Although our results do not allow a full understanding of the volcano/intrusion parameters that control the development of the different deformation patterns detected, the different surface deformation features of each pattern (Figs 2, 3 and 4) are distinctive enough to allow use them for the forecasting of possible eruption scenarios (Fig. 6). So in practical, although the likelihood of the intrusion being located in the landslide, or under it, cannot be predicted, our results show that once one such case develops, the characteristic structural pattern gives a strong indication of the probable scenario that may follow. This opens the possibility that there may be early signs to help distinguish the two possible outcomes (blast vs. no-blast eruptive scenarios) earlier.

Firstly, our results show that the comparison of model surface deformation features with actual observations of the surface faulting and flank deformation during the unrest start by volcano deformation monitoring systems (GPS or InSAR) could be used to identify the volcano deformation pattern produced by the intrusive episode (Fig. 6). Specifically, the monitoring data could thus allow an early detection of the development of a type-3 deformation pattern, indicating the hazard zone for a possible lateral collapse and the likelihood of an associated lateral blast. This potential for predicting the instability and the possible eruptive scenario of a volcano based on its external deformation features has been possible due to the capacity of our novel MDCT methodology for observing simultaneously both the experiment surface and the internal deformation structures in 4D.

Specifically, volcano deformation pattern 3 that can induce the edifice lateral collapse, is characterized initially by the development of a large inward-dipping normal fault near the summit area that can be accompanied by opposite flank deformation (Fig. 6) measurable by volcano monitoring systems (GPS or InSAR). If this situation is detected, the zone potentially affected by a lateral collapse can be determined, and early on, a vertical eruption can be expected if there is volcano collapse (Fig. 6: Early Collapse). However if volcano faulting and surface deformation (subsidence and bulging) begin to develop oblique to the first major fault (Fig: 4: t2), then magma has probably risen over the listric fault (the potential main slip surface; Fig. 4: t2). This changes the hazard assessment, because then on collapse, a laterally directed blast should be expected (Fig. 6: Late Collapse). Our results therefore highlight the role that a deformation monitoring system can play in evaluating a possible lateral collapse and the related eruptive scenarios, taking into account the structural patterns detected by our modelling.

## Methods

### Scaling

The model has been geometrically, kinematically and dynamically scaled^10,20^. To ensure that our models are similar to nature, we define the model scale factors: the ratio between characteristic parameters in the both the model and the nature. For example, length ratio is L* = L_M_/L_N_, where the subscripts M and N refer to model and nature. We made a purely mechanical model so the three basic dimensions to define their scale ratios are length [L], time [T] and mass [M] (refs^10,20^). All the other parameters involved (density, stress, cohesion, viscosity, flow rate) are derived from those three. All the parameters with the same units must have the same scaling ratio, and the ratio of derived parameters must observe the ratio of the involved basic parameters.

We simulated the volcano using an elasto-plastic granular material: a mixture (1–4) of gypsum plaster and quartz sand (grain size of 50% of 125 µm and 50% of 250 µm). Shear-ring tests^21^ provide material mechanical properties: cohesion of around 50–100 Pa and a friction angle of 36–37°. Magma was simulated using a common well-characterized Newtonian fluid (Lyle’s Golden Syrup^22^).

Due to space constraints in the X-ray scanner (see Experimental Setup and Monitoring System), we selected a geometric scale ratio of 1:10,000: [L]* = 1 × 10^−4^. Our analogue volcano is 11 cm-high so we are simulating volcanoes 1100 m-high, typical of a mature simple stratovolcano^23^. Our 1 cm in diameter tube simulates a cylindrical volcanic conduit similar to some natural examples of viscous intrusions into stratovolcanoes^24^, with a diameter 100 m-wide in agreement with conduit diameters of natural viscous magma intrusions (e.g., St Helens 1980; ref.^25^). The produced surface deformations of several millimetres correspond to tens of meters of natural deformation similar to those measured in several volcanoes^5^. The resulting syrup intrusions develop cylindrical, massive and cup-shaped geometries a few millimetres of thickness and width, which agree with the size and shapes of described intermediate-felsic magmatic plugs and intrusive bodies at eroded stratovolcanoes^26,27^ or the modelled laccolith intruded at the base of Cordón Caulle volcano at 2011 (ref.^28^).

The second basic parameter [M] has been derived from the density [M L^−3^] of the used materials. Our sand-plaster mixture has a density of 1320 kg m^−3^. Considering a mean density value for the volcano of 2500 kg m^−3^ (ref.^29^), we obtained a density ratio ρ* = 5.3 × 10^−1^. We combine the parameters L* and ρ* to deduce our mass ratio (M*):

1 $${{\rm{\rho }}}^{\ast }=5.3\times {10}^{-1};\,{{\rm{M}}}^{\ast }\cdot {{\rm{L}}}^{\ast -3}=5.3\times {10}^{-1};\,{{\rm{M}}}^{\ast }=5.3\times {10}^{-13}$$

As the density of natural volcano edifices is not well constrained, we checked the influence of the plausible variations of that value in our scaling. If we introduce a range of ± 100 kg m^−3^ in volcano density value^29^ then the mass ratio range M* = 5.5 × 10^−13^ – 5.1 × 10^−13^. For that range of values of ρ*, and a density for Golden Syrup at our working temperatures (23–24 °C) of 1440 kg m^−3^ (ref.^22^), our scaling results in a density for the simulated magma between 2600–2800 kg m^−3^, consistent with published values for the density of intermediate magmas^30^.

Time is usually the most challenging parameter to properly scale in these models. Previous authors^9,13^ have derived it from comparison between the time (or velocity) of their models and that of the modelled processes, or by using the ratio viscosity*/stress*. In this work we propose a different approach for time scaling using the principle of relationship between variables. Since we do our experiments with natural gravity, g* = 1, and g units are [L T^−2^], we can deduce the time ratio for our experiments combining h* and g*:

2 $${{\rm{g}}}^{\ast }=1;\,{{\rm{L}}}^{\ast }\cdot {{\rm{T}}}^{\ast -2}=1;\,{{\rm{L}}}^{\ast }={1\times 10}^{-4};\,{{\rm{T}}}^{\ast }={1\times 10}^{-2}$$

Using L*, T* and M* we can deduce the ratios for all the other involved parameters.

The ratios for the parameters which units are Pa (Pa = kg m^−1^ s^−2^), as the stress σ, are:

3 $${{\rm{\sigma }}}^{\ast }={{\rm{M}}}^{\ast }\cdot {{\rm{L}}}^{\ast -1}\cdot {{\rm{T}}}^{\ast -2};\,{{\rm{\sigma }}}^{\ast }=5{.3\times 10}^{-13}\times {({1\times 10}^{-4})}^{-1}\times {({1\times 10}^{-2})}^{-2}=5.3\times {10}^{-5}$$

and taken into account the possible range for ρ*, then σ* = 5.5 × 10^−5^ – 5.1 × 10^−5^. This ratio must be also observed by the cohesion (C) of the elasto-plastic materials (C_M_ = 50–100 Pa). Considering the range of cohesion ratio deduced we are simulating a volcano edifice with a cohesion of C_N_ ≈ 0.9–2 MPa, similar to those proposed for fractured volcanic rock masses^29^.

As our experiments are rate-dependent, viscosity is interrelated to the other parameters involving time (e.g. flow rate). In order to verify that our experiments are properly scaled for representative intrusion processes of viscous magma bodies in stratovolcanoes, we check if we are simulating magma intrusions with flow rates (Q_N_) and viscosities (µ_N_) values consistent with real examples, fixing h*, σ* and µ_M_:

4 $${\mu }_{{\rm{N}}}=({{\rm{Q}}}_{{\rm{M}}}\,\cdot \,{\mu }_{{\rm{M}}}){/({\rm{L}}}^{\ast 3}\,\cdot \,{{\rm{\sigma }}}^{\ast }{\cdot {\rm{Q}}}_{{\rm{N}}})$$

Golden Syrup at our working temperatures has a viscosity of µ_M_ = 23–37 Pa s (ref.^22^). Natural examples of viscous magma intrusions in stratovolcanoes usually have flow rates between 1 and <100 m^3^ s^−1^ (refs^31,32^). Using the above equation we can observe that for our range of Q_M_ (1–3 cm^3^ min^−1^) for Q_N_ = 1 m^3^ s^−1^ we are simulating magmas with a viscosity of 1–1.5 × 10^10^ Pa s, and for Q_N_ = 100 m^3^ s^−1^ magmas with a viscosity of 1–1.5 × 10^8^ Pa s, similar to those deduced for viscous intrusions deforming stratovolcanoes^33,34^. For example, for an experiment with a Q_M_ = 3 cm^3^ min^−1^ we can simulate the intrusion of a magma with a viscosity of 1 × 10^9^ Pa s at a flow rate of 30 m^3^ s^−1^, the same values proposed for the 1980 cryptodome intruded at Mount St Helens^34,35^.

The desired range of flow rates between ≈ 1 and 3 cm^3^ min^−1^ are produced when the syrup container is located at heights between 0.85 and 1.65 m above the top of the surface of the table. The resulting pressures produced by the syrup at the base of the volcano for those heights in our device (i.e., the hydrostatic pressure minus the viscous drag) are about 5–15 × 10^3^ Pa. These values correspond to natural magma pressures of about 100–250 MPa, higher than those usually supposed occurring at the base of stratovolcanoes (around 10 MPa due to the tensile strength of volcanic rock masses; e.g., ref.^36^) or for example those calculated at the volcano conduit during the intrusion in Soufrière Hills volcano at 1997 (40–100 MPa; ref.^36^). However, since brittle behaviour is not strain-rate dependant and the angles of the faults produced in our experiments agree with the predictions of a Mohr-Coulomb failure criterion for the sand-plaster mixture, we consider that the high-scaled pressure made by the syrup is not negatively influencing the brittle faulting patterns produced during the experiments.

Finally, we also check if the intrusion time of our experiments is properly scaled, obtaining that for magma viscosities of 1 × 10^8^ − 1 × 10^10^ Pa s we are simulating intrusions several days long, consistent with natural examples: e.g., the cryptodome intrusion at St Helens at 1980 (58 days^35^) is properly scaled in one experiment of around 45 minutes long.

### Experimental setup and monitoring system

The device employed to monitor our experiments was a 64-detector row MDCT Scanner Siemens Definition AS 64 (Siemens Healthcare, Erlangen, Germany) at the Hospital Rey Juan Carlos (Móstoles, Madrid). The scan parameters were: Detector configuration 64 × 0.6 mm, slice thickness 1 mm, pitch 0.35, FOV 31 × 31 cm. All images were reconstructed applying a filtered backprojection with two Kernels, a standard one B20, and a sharp one B75.

We tested the feasibility of scanning the experiments with a continuous syrup flow, checking if scanner platform displacement introduced any undesirable influence, by comparing results (syrup flow rates and volcano surface morphologies) between experiments performed statically (34 similar experiments made at University Rey Juan Carlos laboratory) and at different platform velocities. We observed that a scanning period of 26 seconds (a slower table velocity than the typical for medical applications) did not disturb the experiments.

During each scan of the experiment, sequential slices were obtained perpendicular to the mobile platform. Each slice is represented as an image of 512 × 512 pixels (0.6 mm/pixel) giving quantitative information (at 256 grey values) on density distribution of the scanned volume. Each slice is 1 mm thick, so our final volume is formed by around 300 slices.

Image processing software OsiriX^37^ (open-source software: www.osirixviewer.com) was used to process DICOM images (www.dicom.nema.org) in order to generate multiplanar reconstruction (MPR) and volumetric reconstruction (Volume Rendering). The two different Kernel filters allowed us to better assess different attenuations inside the volcano and detection of small fractures. We have produced Digital Elevation Models (DEM) of the volcanoes, by extracting the volcano surface at obj format at Osirix and then process it with MOVE (Midland Valley) software (https://www.mve.com/software/move) to produce a volcano DEM in ASCII format.

Additionally, we have made 34 experiments at the University Rey Juan Carlos laboratory for comparison and control, where the experiments have been monitored using a Kinect v2 device, obtaining both surface images and distance data^38^ which have been used to produce faulting maps and DEMs of the volcano.

## References

[1] Carrasco-Núñez, G., Siebert, L. & Capra, L. Hazards from volcanic avalanches. *In Horizons in Earth Science Research* (eds Veress, B. & Szigethy, J.) **3**, 199–227 (Nova Science Publishers, 2011).

[2] Samaniego P (2015). The historical (218 ± 14 aBP) explosive eruption of Tutupaca volcano (Southern Peru). Bull. Volcanol..

[3] Siebert L, Glicken H, Ui T (1987). Volcanic hazards from Bezymianny-and Bandai-type eruptions. Bull. Volcanol..

[4] Belousov A, Voight B, Belousova M (2007). Directed blasts and blast-generated pyroclastic density currents: a comparison of the Bezymianny 1956, Mount St Helens 1980, and Soufrière Hills, Montserrat 1997 eruptions and deposits. Bull. Volcanol..

[5] Lipman, P. W., Moore, J. G., & Swanson, D. A. In *The 1980 eruptions of Mount St. Helens, Washington* (eds Lipman, P. W. & Mullineaux, D. R.) 143–156 (U.S. Geol. Surv. Prof. Pap., Washington, 1981).

[6] Gorshkov GS (1959). Gigantic eruption of the volcano Bezymianny. Bull. Volcanol..

[7] Donnadieu F, Kelfoun K, van Wyk de Vries B, Cecchi E, Merle O (2003). Digital photogrammetry as a tool in analogue modelling: applications to volcano instability. J. Volcanol. Geotherm. Res..

[8] Belousov A, Belousova M, Voight B (1999). Multiple edifice failures, debris avalanches and associated eruptions in the Holocene history of Shiveluch volcano, Kamchatka, Russia. Bull. Volcanol..

[9] Donnadieu F, Merle O (1998). Experiments on the indentation process during cryptodome intrusions: new insights into Mount St. Helens deformation. Geology.

[10] Galland, O., Holohan, E., van Wyk de Vries, B. & Burchardt, S. In *Laccoliths, Sills and Dykes - Physical geology of shallow level magmatic systems* (eds Breitkreutz, C. & Rocchi, S.) 1–68 (Springer, Advances in Volcanology, Berlin, 2015).

[11] Elsworth D, Voight B (1995). Dike intrusion as a trigger for large earthquakes and the failure of volcano flanks. Jour. Geophys. Res.: Solid Earth.

[12] Adam J, Klinkmüller M, Schreurs G, Wieneke B (2013). Quantitative 3D strain analysis in analogue experiments simulating tectonic deformation: Integration of X-ray computed tomography and digital volume correlation techniques. J. Struct. Geol..

[13] Mathieu L, van Wyk de Vries B, Holohan EP, Troll VR (2008). Dykes, cups, saucers and sills: Analogue experiments on magma intrusion into brittle rocks. Earth Planet. Sci. Lett..

[14] Donnadieu, F. Déstabilisation des édifices volcaniques par les cryptodômes: modélisation analogique et approche numérique (Doctoral dissertation, 2000).

[15] Cecchi E, van Wyk de Vries B, Lavest JM (2004). Flank spreading and collapse of weak-cored volcanoes. Bull. Volcanol..

[16] Wooller L, van W de Vries B, Murray JB, Rymer H, Meyer S (2004). Volcano spreading controlled by dipping substrata. Geology.

[17] Delcamp A, van W de Vries B, James B, Gailler MR, Lebas LS (2012). E. Relationships between volcano gravitational spreading and magma intrusion. Bull. Volcanol..

[18] van Wyk de Vries B, Francis PW (1997). Catastrophic collapse at stratovolcanoes induced by gradual volcano spreading. Nature.

[19] Siebert L (1984). Large volcanic debris avalanches: characteristics of source areas, deposits, and associated eruptions. J. Volcanol. Geotherm. Res..

[20] Merle O (2015). The scaling of experiments on volcanic systems. Front. Earth Sci..

[21] Klinkmüller M, Schreurs G, Rosenau M, Kemnitz H (2016). Properties of granular analogue model materials: A community wide survey. Tectonophysics.

[22] Beckett FM, Mader HM, Phillips JC, Rust AC, Witham F (2011). An experimental study of low-Reynolds-number exchange flow of two Newtonian fluids in a vertical pipe. J. Fluid Mech..

[23] Grosse P, van W de Vries B, Petrinovic B, Euillades IA, Alvarado PA (2009). G. E. Morphometry and evolution of arc volcanoes. Geology.

[24] Albino, F., Pinel, V., Massol, H., & Collombet, M. Conditions for detection of ground deformation induced by conduit flow and evolution. *Jour. Geophys. Res.: Solid Earth* **116**(B6) 10.1029/2010JB007871 (2011).

[25] Dobran F (1992). Nonequilibrium flow in volcanic conduits and application to the eruptions of Mt. St. Helens on May 18, 1980, and Vesuvius in AD 79. J. Volcanol. Geotherm. Res..

[26] Taylor, E. M. Field geology of SW Broken Top quadrangle, Oregon (No. 2). State of Oregon, Dept. of Geology and Mineral Industries (1978).

[27] Lipman PW (1968). Geology of the summer coon volcanic center, eastern San Juan Mountains, Colorado. Colo. Sch. Mines Q.

[28] Castro JM (2016). Rapid laccolith intrusion driven by explosive volcanic eruption. Nature Communications.

[29] Seisdedos J, Ferrer M, de Vallejo LG (2012). Geological and geomechanical models of the pre-landslide volcanic edifice of Güímar and La Orotava mega-landslides (Tenerife). J. Volcanol. Geotherm. Res..

[30] Spera, F. J. In *Encyclopedia of volcanoes* (eds Sigurdsson, H., Houghton, B., Rymer, H., Stix, J. & McNutt, S.) 171–190 (Academic Press, San Diego, 2000).

[31] Pallister JS (2013). Merapi 2010 eruption—Chronology and extrusion rates monitored with satellite radar and used in eruption forecasting. J. Volcanol. Geotherm. Res..

[32] Kozono T (2013). Magma discharge variations during the 2011 eruptions of Shinmoe-dake volcano, Japan, revealed by geodetic and satellite observations. Bull. Volcanol..

[33] Pinkerton H, Stevenson RJ (1992). Methods of determining the rheological properties of magmas at sub-liquidus temperatures. J. Volcanol. Geotherm. Res..

[34] Lavallée Y, Hess KU, Cordonnier B, Dingwell DB (2007). Non-Newtonian rheological law for highly crystalline dome lavas. Geology.

[35] Moore, J. G., & Albee, W. C. In *The1980 eruptions of Mount St. Helens, Washington* (eds Lipman, P. W. & Mullineaux, D. R.) 123–134 (U.S. Geol. Surv. Prof. Pap., Washington, 1981).

[36] Green, D. N., Neuberg, J., & Cayol, V. Shear stress along the conduit wall as a plausible source of tilt at Soufrière Hills volcano, Montserrat. *Geophys. Res. Lett*. **33**(10), 10.1029/2006GL025890 (2006).

[37] Rosset A, Spadola L, Ratib O (2004). OsiriX: an open-source software for navigating in multidimensional DICOM images. J. Digit. Imaging.

[38] Tortini R, Bonali FL, Corazzato C, Carn SA, Tibaldi A (2014). An innovative application of the Kinect in Earth sciences: quantifying deformation in analogue modelling of volcanoes. Terra Nova.

